# High expression of Rab25 contributes to malignant phenotypes and biochemical recurrence in patients with prostate cancer after radical prostatectomy

**DOI:** 10.1186/s12935-017-0411-0

**Published:** 2017-04-11

**Authors:** Chunhui Hu, Beibei Chen, Yibin Zhou, Yuxi Shan

**Affiliations:** 1 Department of Urology, The Second Affiliated Hospital of Soochow University, Suzhou, 215004 China; 2 Department of Intensive Care Unit, Huai’an First People’s Hospital, Huai’an, 223300 Jiangsu China

**Keywords:** Prostate cancer, Ras-related protein 25, Biochemical recurrence, Malignant progression

## Abstract

**Background:**

Ras-related protein 25 (Rab25) functions either as an oncogene or a tumor suppressor with a cancer type-dependent manner. We aimed to investigate clinical significance of Rab25 in prostate cancer (PCa).

**Methods:**

Quantitative real-time polymerase chain reaction, Western blot and immunohistochemistry were respectively performed to detect Rab25 mRNA and protein expression in PCa and adjacent non-cancerous prostate tissues. Receiver-operating characteristic curve analysis was used to evaluate predictive diagnostic value of Rab25. Associations of Rab25 expression with various clinicopathological characteristics and biochemical recurrence-free survival of PCa patients were statistically evaluated. In vitro, PCa cell proliferation was assessed by CCK-8 assay, and the cell migration and invasion activities were evaluated by Transwell assay, following the transfection of Rab25 small interfering RNA.

**Results:**

Ras-related protein 25 mRNA and protein expression in PCa tissues were both significantly higher than adjacent non-cancerous prostate tissues (both P < 0.001). The area under the curve of Rab25 immunoreactive score (IRS) was 0.896 (P < 0.001) with 74.0% sensitivity and 95.0% specificity. High Rab25 IRS was significantly associated with high Gleason score (P = 0.02) and distant metastasis (P = 0.01). PCa patients with high Rab25 IRS had shorter overall and biochemical recurrence-free survivals than those with low Rab25 IRS (both P < 0.001). Cox regression analysis identified Rab25 as an independent biomarker for both overall and biochemical recurrence-free survivals of PCa patients. By exploring its activities in vitro, Rab25 downregulation was found to inhibit PCa cell proliferation, migration and invasion.

**Conclusions:**

High expression of Rab25 may contribute to malignant progression and biochemical recurrence of PCa patients after radical prostatectomy.

**Electronic supplementary material:**

The online version of this article (doi:10.1186/s12935-017-0411-0) contains supplementary material, which is available to authorized users.

## Background

Prostate cancer (PCa), as one of the most frequent malignancies in male who are older than 50 years of age, represents the second leading cause of death from cancer among men [1]. Statistically, there are more than 200,000 newly diagnosed cases with PCa annually throughout the world [2]. Although a number of PCa patients can be diagnosed at an early stage and low-risk disease may exert no impact on patients’ life expectancy, it is a great challenge to develop effective strategies to treat metastasis and biochemical recurrence of PCa which cannot be treated by surgery or adjuvant therapies [3]. Various clinicopathological characteristics, such as serum prostate specific antigen (PSA) level, clinical stage and Gleason score (GS), offer a contribution to make a treatment decision before the administration of an intervention [4]. However, it often leads to controversial results due to the lack of reliable molecular biomarkers which are closely correlated with the clinicopathological characteristics of PCa and can be used to efficiently distinguish the patients with high-risk PCa from insignificant disease [5]. Therefore, it is urgently needed to identify novel and effect diagnostic and prognostic biomarkers for this cancer.

Ras-related protein 25 (Rab25, also named as Rab11c and Catx-8), together with Rab11a and Rab11b, was identified in 1993 as a member of the Rab small GTPase family [6]. Rab25 functions as an intracellular transport protein which plays a crucial role in selectively regulating apical recycling and/or transcytosis pathways [7]. Accumulating studies have shown the aberrant expression of Rab25 in various human cancers and also indicated its associations with carcinogenesis, cancer progression and patients’ outcome [8–16]. Rab25 is frequently amplified in head and neck tumors [8], gastric cancer [9], ovarian cancer [10], cervical cancer [11], bladder cancer [12] and renal cancer [13], but is downregulated in esophageal cancer [14], breast cancer [15] and colorectal cancer [16]. Functionally, it acts either as an oncogene or a tumor suppressor with a cancer-specific manner.

Recently, Kessler et al. [17] observed the overexpression of Rab25 in acinar adenocarcinoma of the prostate obtained from nine random patients by immunohistochemistry. To determine the expression patterns of Rab25 protein and gene in PCa tissue specimens, and to investigate its clinical significance, quantitative real-time polymerase chain reaction and Western blot analyses were respectively performed to detect the expression of Rab25 mRNA and protein using 20 pairs of PCa and adjacent non-cancerous prostate tissues. Immunohistochemistry was performed to examine the subcellular localization and the expression of Rab25 protein in 100 pairs of PCa and adjacent non-cancerous prostate tissues. Receiver-operating characteristic curve analysis was used to evaluate predictive diagnostic value of Rab25. Chi square test was then used to evaluate the associations between Rab25 expression and various clinicopathological characteristics. Kaplan–Meier and Cox proportional hazards regression models were used to assess the prognostic value of Rab25 expression in PCa patients. In vitro, PCa cell proliferation was assessed by CCK-8 assay, and the cell migration and invasion activities were evaluated by Tanswell assay, following the transfection of Rab25 small interfering RNA (siRNA).

## Methods

### Patients and tissue samples

Our study was approved by the Ethics Committees of the Second Affiliated Hospital of Soochow University and Huai’an First People’s Hospital. Informed consent was obtained from all patients prior to the sample collection based on the guidelines of the Second Affiliated Hospital of Soochow University and Huai’an First People’s Hospital. All tissue specimens were handled and made anonymous according to the ethical and legal standards.

For quantitative real-time polymerase chain reaction and Western blot analyses, 20 pairs of PCa and adjacent non-cancerous prostate tissues were obtained from 20 patients with primary PCa diagnosed at the Department of Urology Surgery in the Second Affiliated Hospital of Soochow University and Huai’an First People’s Hospital between August 2014 and July 2015. Ages of these patients varied from 42 to 86 years (median, 66 years). All the specimens were biopsy materials and frozen in liquid nitrogen immediately, then the samples were stored at −80 °C for RNA or protein extraction.

For immunohistochemistry analysis, a total of 100 paraffin-embedded PCa and adjacent non-cancerous prostate tissues were obtained from 100 patients with primary PCa diagnosed at the Department of Urology Surgery in the Second Affiliated Hospital of Soochow University and Huai’an First People’s Hospital between January 2000 and December 2005. Ages of these patients varied from 46 to 82 years (median, 66 years). After radical prostatectomy, all 100 PCa patients received a follow-up of 60 months. The follow-up information was obtained via a telephone or questionnaire and was updated every 3 months. Biochemical recurrence was defined by an elevation of serum PSA level at 3 consecutive measurements. The overall survival time was defined as the time from day of surgery to the day of death. The biochemical recurrence-free survival time was defined as the time from day of surgery to the day of biochemical recurrence. Patients who died from other diseases or accident were excluded from case collection.

All the PCa patients had not received chemotherapy, radiation therapy, or androgen deprivation before surgery. The diagnosis was confirmed by two pathologists who were blinded to the clinical information. Tumor and clinical stages were defined according to the criteria of the 2002 TNM Classification [18] and the Gleason system [19]. The clinical characteristics of all PCa patients, including age at diagnosis, preoperative serum PSA level, clinical stage, GS and distant metastasis, were summarized in Table 1.

**Table 1:** Clinicopathologic characteristics of all PCa patients

Characteristics	Experiment type	
	Immunohistochemistry	Quantitative real-time polymerase chain reaction/western blot
Prostate cancer (cases)	100	20
Age (years)		
≤66	38	8
>66	62	12
Preoperative serum PSA levels (ng/ml)		
<4	20	5
≥4	80	15
Clinical stage		
T1 + T2	52	12
T3 + T4	48	8
Gleason score		
<7	52	11
≥7	48	9
Metastasis	10	2
Adjacent non-cancerous prostate tissue (cases)	100	20

### Cell culture and transfection

One human PCa cell line LNCaP and one normal human prostate epithelial cell line PrEC were purchased from American Type Culture Collection (ATCC). The cells were cultured in RPMI-1640 mediums with 10% fetal bovine serum (FBS, Invitrogen, USA), 100 units/ml penicillin, and 100 μg/ml streptomycin, and were incubated at 37 °C in a humidified atmosphere of 5% CO_2_.

To knockdown Rab25 expression, LNCaP cells were transfected with 100 nM Rab25 siRNA (ThermoFisher, USA) using Lipofectamine 2000 (Invitrogen, USA) according to the manufacturer’s instructions. Transfection efficiency was evaluated at 24 h after the transfection.

### Quantitative real-time polymerase chain reaction

Expression levels of Rab25 mRNA in 20 pairs of PCa and adjacent non-cancerous prostate tissues were detected by quantitative real-time polymerase chain reaction. Total RNAs were isolated using TRIzol Reagent (Life Technologies Corporation, Carlsbad, CA, USA) according to the users’ protocol. Then, cDNA was synthesized by RNA reverse transcription using PrimeScript RT-PCR Kit (Takara Bio, TBUSA, formerly Clontech Laboratories, Inc., Mountain View, CA, USA) according to the users’ protocol. The real-time polymerase chain reaction was performed using the Applied Biosystems 7500 Fast Real-Time PCR System (Applied Biosystems, Foster City, California, USA). GAPDH was used as an internal reference gene for Rab25. The primer sequences were as following: for Rab25 mRNA: forward 5′- TCG CTG AAA ACA ATG GAC TGC TCT T -3′, reverse 5′- ATT GGT CCG GAT GCT GTT CTG TCT CT -3′; for GAPDH: forward 5′- ATG GAA ATC CCA TCA CCA TCT T -3′, reverse 5′- CGC CCC ACT TGA TTT TGG -3′. Relative quantification of target mRNA expression was evaluated using the comparative cycle threshold (CT) method. Each sample was examined in triplicate.

### Western blot analysis

Expression levels of Rab25 protein in 20 pairs of PCa and adjacent non-cancerous prostate tissues were detected by Western blot analysis. Proteins were extracted from tissue specimens using radio-immunoprecipitation assay buffer. The protein concentration was determined by the Bradford assay (Bio-Rad, California, USA) using a BCA Protein Assay Kit (Beyotime, Haimen, China). The protein samples were separated by 10% sodium dodecyl sulfate polyacrylamide gel electrophoresis (SDS-PAGE) and transferred onto polyvinylidene difluoride membranes (Qiagen China Co., Ltd.). Then, the membranes were incubated with the primary antibodies: anti-Rab25 (dilution 1:500, Mouse monoclonal, #ab106175, Abcam Inc, MA, USA) and anti-GAPDH (dilution 1:1000, Mouse monoclonal, #ab8245, Abcam Inc, MA, USA), after blocking with 8% milk in phosphate-buffered saline (PBS; pH 7.5). After that, the membranes were incubated with the appropriate horseradish peroxidase (HRP)-conjugated secondary antibodies (dilution 1:1000, # ab131368, Abcam Inc, MA, USA) after the incubation at 4 °C overnight. A Western Bright ECL kit (Advansta, Menlo Park, CA) was used for the detection of HRP in the antigen–antibody complex, and protein bands were detected by using an enhanced chemiluminescence system (Amersham Bioscience, Piscataway, NJ). Each sample was examined in triplicate.

### Immunohistochemistry

Expression pattern and subcellular localization of Rab25 protein in 100 pairs of PCa and adjacent non-cancerous prostate tissues were examined by immunohistochemistry analysis. Paraffin-embedded sections were deparaffinized in xylene and rehydrated with different concentrations of alcohol. The endogenous peroxidase activities was blocked using 0.3% hydrogen peroxide for 30 min. Then, the sections were incubated with the primary antibody to Rab25 protein (dilution 1:250, Mouse monoclonal, #ab106175, Abcam Inc, MA, USA) at 4 °C overnight. After that, the sections were incubated with HRP-conjugated secondary antibody (dilution 1:500, #ab131368, Abcam Inc, MA, USA) for 1 h at room temperature. After washing with phosphate buffered saline (PBS), the sections were incubated with 3, 3′-diaminobenzidine tetrahydrochloride (DAB), and counter-stained with hematoxylin, dehydrated, and coverslipped. For the negative control, the primary antibody was replaced by PBS. For the positive control (Additional file 1: Figure S1), the renal cell carcinoma (RCC) tissues were used, since a previous study of Liu et al. [20] observed the over-expression of Rab25 protein in RCC tissues.

Two independent pathologists, who were both blinded to the patient background, were invited to evaluate immunohistochemical results of Rab25 protein based on staining intensity and positive cells’ percentage as described in previous studies [21]. Briefly, staining intensity was scored as follows: negative-‘0’, weakly positive-‘1’, moderately positive-‘2’, and strongly positive-‘3’. The percentage of Rab25-positive cells was scored as 0%- ‘0’, 1–25%-‘1’, 26–50%-‘2’, and >50%-‘3’. The immunoreactive score (IRS) of Rab25 protein for each tissue section was calculated by multiplying the intensity and the percentage scores, and ranged from 0 to 9. For statistical analysis, all 100 PCa patients were divided into two groups using the median value of Rab25 protein as a cutoff point: low Rab25 group (score 0–4.0), and high Rab25 group (score 4.0–9.0).

### Cell proliferation assay

Cell proliferation of LNCaP cells with or without Rab25 siRNA transfection was assessed using the Cell Counting Kit-8 (CCK-8, Dojindo, Kumamoto, Japan) per the manufacturer’s instructions. The number of viable cells was measured with the absorbance at 450 nm.

### Cell invasion and migration assays

Cell invasion and migration abilities of LNCaP cells with or without Rab25 siRNA transfection were evaluated by the transwell assay using the transwell chambers with or without coated-Matrigel (8 μm, 24-well insert; Corning, Lowell, MA, USA) per the manufacturer’s instructions. PCa cells that invaded or migrated into the lower chambers were fixed with methanol, stained with crystal violet, and counted in six random fields.

### Statistical analysis

All statistical analyses were performed using the software of SPSS (version 11.0, SPSS Inc, IL, USA). Data were shown as mean ± standard deviation. P values less than 0.05 were considered statistically significant. The differences between two groups were analyzed using Student’s t test. Receiver operating characteristic (ROC) curves were established to evaluate the diagnostic value of Rab25 expression. The associations of Rab25 expression with PCa patients’ clinicopathologic characteristics were evaluated using the χ^2^ test. The correlation between Rab25 mRNA and protein expression in PCa tissues was determined by Spearman Correlation analysis. The Kaplan–Meier survival curves were used to determine any significant relationships between Rab25 protein expression, and overall survival and biochemical recurrence-free survival.

## Results

### Overexpression of Rab25 mRNA and protein in human PCa tissues and cells

**Fig. 1:**
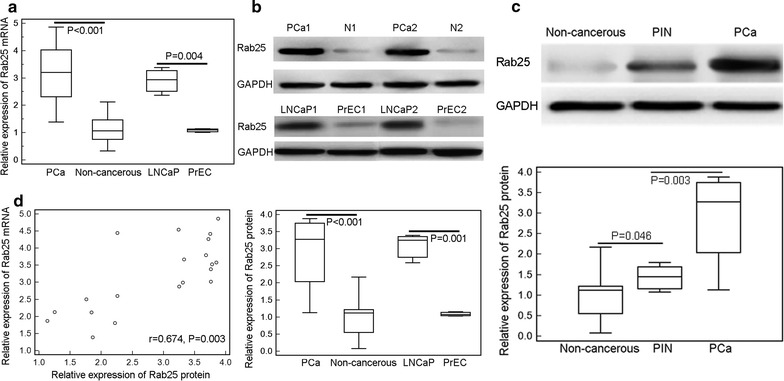
Overexpression of Rab25 mRNA and protein in human PCa tissues and cells. **a** Expression levels of Rab25 mRNA in 20 pairs of PCa and adjacent non-cancerous prostate tissues, LNCaP and PrEC cells were detected by quantitative real-time polymerase chain reaction; **b** expression levels of Rab25 protein in 20 pairs of PCa and adjacent non-cancerous prostate tissues, LNCaP and PrEC cells were detected by Western blot analysis; **c** expression levels of Rab25 mRNA in PCa tissues were positively correlated with those of Rab25 protein in PCa tissues. *Note* ‘N’ refers to adjacent non-cancerous prostate tissues

Expression levels of Rab25 mRNA were both significantly higher in PCa tissues and LNCaP cells than those in adjacent non-cancerous prostate tissues and PrEC cells (cancer vs. normal: 3.19 ± 1.03 vs. 1.15 ± 0.51, P < 0.001; LNCaP vs. PrEC: 2.90 ± 0.51 vs. 1.09 ± 0.07, P = 0.004; Fig. 1a), which was consistent with the data on Rab25 protein obtained from Western blot analysis (cancer vs. normal: 2.89 ± 0.96 vs. 0.95 ± 0.50, P < 0.001; LNCaP vs. PrEC: 3.08 ± 0.43 vs. 1.09 ± 0.07, P = 0.001, Fig. 1b). In addition, we also detected the expression levels of Rab25 protein in only five PIN tissues using Western blot analysis. As shown in Fig. 1c, Rab25 protein expression in PIN tissues was significantly higher than non-cancerous prostate tissues (PIN vs. non-cancerous: 1.44 ± 0.30 vs. 0.95 ± 0.50, P = 0.046), but were dramatically lower than those in PCa tissues (PIN vs. PCa: 1.44 ± 0.30 vs. 2.89 ± 0.96, P = 0.003). Due to the small cohort of PIN group, the future validation using a large cohort is required. Moreover, the expression levels of Rab25 mRNA in PCa tissues were positively correlated with those of Rab25 protein in PCa tissues (r = 0.674; P = 0.003; Fig. 1d).

**Fig. 2:**
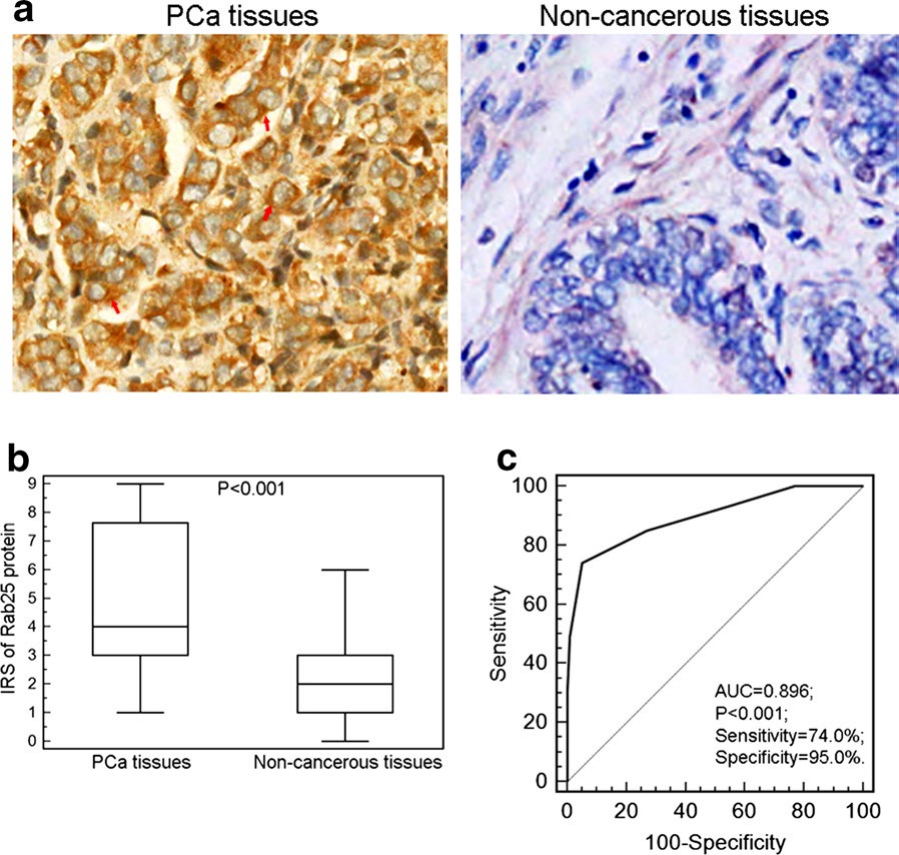
Immunohistochemical results of Rab25 protein in human PCa tissues and adjacent non-cancerous prostate tissues. **a** Immunostainings of Rab25 protein PCa and adjacent non-cancerous prostate tissues. **b** Statistically, the IRS of Rab25 protein showed a significant increase in PCa tissues compared with adjacent non-cancerous prostate tissues (cancer vs. normal: 5.43 ± 2.75 vs. 1.65 ± 1.26, P < 0.001). **c** The ROC of Rab25. The area under the curve (AUC) of Rab25 IRS at the cutoff point 4.0 was 0.896 (P < 0.001, 95% confidence interval = 0.845–0.934) with 74.0% sensitivity and 95.0% specificity

Furthermore, the moderately or strongly positive immunostainings of Rab25 protein were observed in the cytoplasm of cancer cells in PCa tissues, while there was only weak or negative immunostainings of Rab25 protein shown in adjacent non-cancerous prostate tissues (Fig. 2a). Statistically, the IRS of Rab25 protein showed a significant increase in PCa tissues compared with adjacent non-cancerous prostate tissues (cancer vs. normal: 5.43 ± 2.75 vs. 1.65 ± 1.26, P < 0.001, Fig. 2b). The area under the curve (AUC) of Rab25 IRS at the cutoff point 4.0 was 0.896 (P < 0.001, 95% confidence interval = 0.845–0.934) with 74.0% sensitivity and 95.0% specificity (Fig. 2c).

### Overexpression of Rab25 associates with aggressive progression of PCa patients

The median value of Rab25 IRS was used as a cutoff point to divide all 100 PCa patients into high-Rab25 (n = 52) and low-Rab25 (n = 48) expression groups. As shown in Table 2, PCa patients with high Rab25 more frequently had high Gleason score (P = 0.01) and positive distant metastasis (P = 0.01) than those with low Rab25 expression. There were no significant associations between Rab25 IRS and patients’ age, preoperative serum PSA levels and clinical stage (all P > 0.05, Table 2).

**Table 2:** Association of Rab25 protein expression with various clinicopathologic characteristics of 100 PCa patients

Clinical variables	High-Rab25 (n, %)	Low-Rab25 (n, %)	P
Age (years)			
≤66	18 (47.4)	20 (52.6)	NS
>66	34 (54.8)	28 (45.2)	
Preoperative serum PSA levels (ng/ml)			
<4	10 (50.0)	10 (50.0)	NS
≥4	42 (52.5)	38 (47.5)	
Clinical stage			
T1 + T2	30 (57.7)	22 (42.3)	NS
T3 + T4	22 (45.8)	26 (54.2)	
Gleason score			
<7	16 (30.8)	36 (69.2)	0.01
≥7	36 (75.0)	12 (25.0)	
Metastasis			0.01
Negative	43 (47.8)	47 (52.2)	
Positive	9 (90.0)	1 (10.0)	

### Prognostic value of Rab25 protein expression in PCa patients

**Fig. 3:**
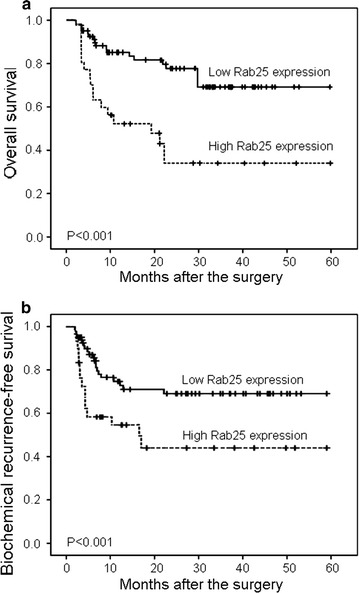
Kaplan-Meier curves of overall survival (**a**) and biochemical recurrence-free survival (**b**) of patients with PCa stratified by the IRS of Rab25 protein

Kaplan–Meier curve analyses revealed that both overall survival and biochemical recurrence-free survival of PCa patients with high Rab25 expression were significantly shorter than those with low Rab25 expression (both P < 0.001, Fig. 3). Moreover, univariate analyses found that overall survival and biochemical recurrence-free survival of PCa patients were both significantly associated with clinical stage (both P < 0.001), Gleason score (both P = 0.01), status of metastasis (both P = 0.006) and Rab25 expression (P = 0.01 and 0.02, respectively).

Furthermore, Cox proportional hazards multivariate analyses of the univariate predictors identified clinical stage (P = 0.006 and 0.01, respectively), Gleason score (both P = 0.03), status of metastasis (both P = 0.01) and Rab25 expression (P = 0.02 and 0.03, respectively) as independent prognostic factors for overall survival and biochemical recurrence-free survival (Table 3).

**Table 3:** Multivariate analysis of the impact of variables on biochemical recurrence-free survival and overall survival in PCa patients

Variable	P	Odds ratio
Overall survival		
Clinical stage		
T1 + T2 vs. T3 + T4	0.006	6.98
Gleason score		
<7 vs. ≥7	0.03	2.82
Metastasis		
Negative vs. positive	0.01	4.16
Rab25 expression		
Low vs. high	0.02	3.09
Biochemical recurrence-free survival		
Clinical stage		
T1 + T2 vs. T3 + T4	0.01	4.09
Gleason score		
<7 vs. ≥7	0.03	2.50
Metastasis		
Negative vs. positive	0.01	4.38
Rab25 expression		
Low vs. high	0.03	2.28

### Knockdown of Rab25 inhibits cell proliferation, invasion and migration of PCa cells in vitro

**Fig. 4:**
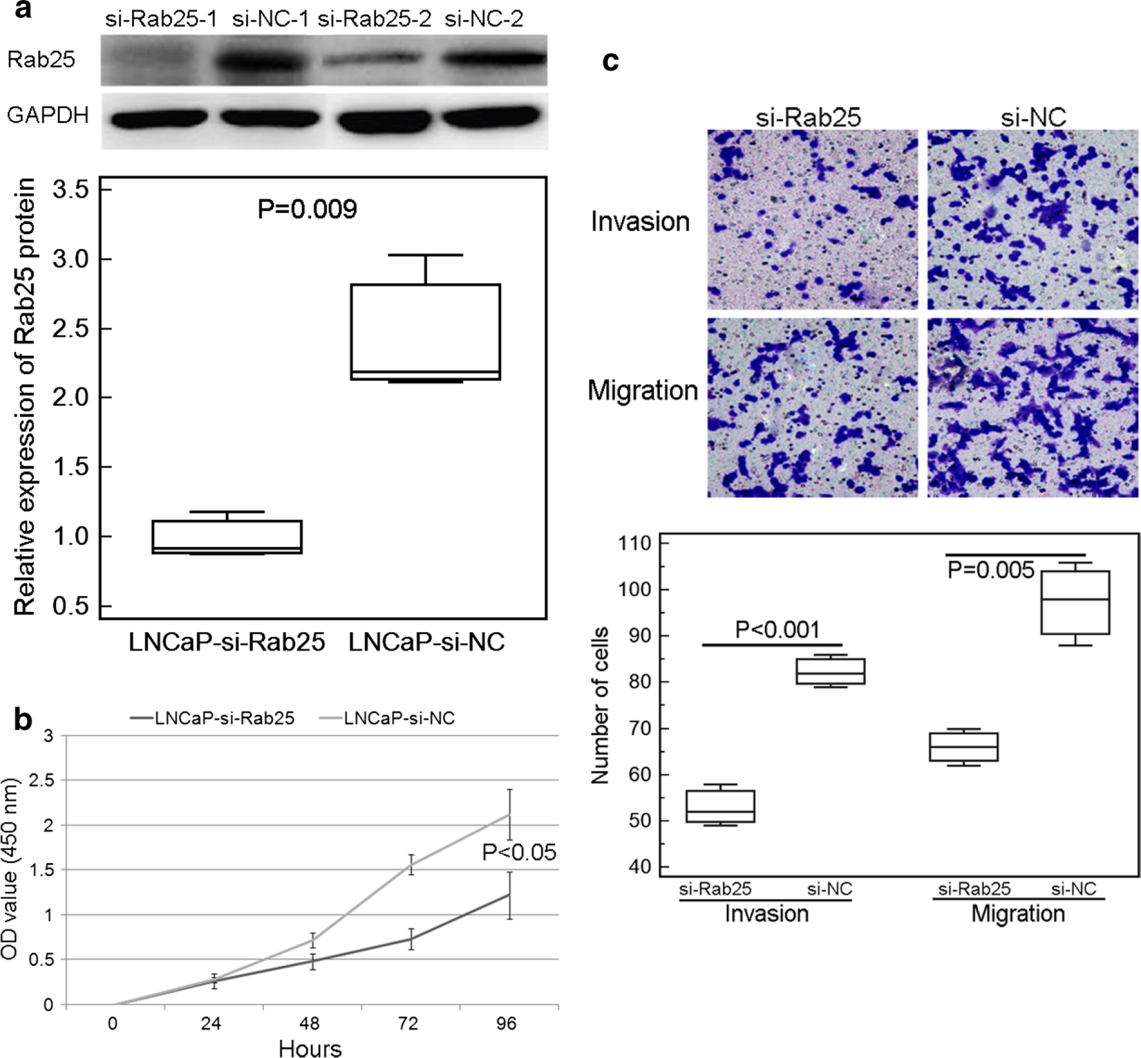
Knockdown of Rab25 inhibits cell proliferation, invasion and migration of PCa cells in vitro. **a** Rab25 protein expression in LNCaP cells transfected with Rab25-siRNA were significantly lower than that transfected with control-siRNA (P = 0.009); **b, c** the cell proliferation, invasion and migration abilities of LNCaP cells with Rab25 siRNA transfection were dramatically lower than those with control siRNA transfection (all P < 0.05). *Note* ‘si-Rab25’ refers to Rab25 siRNA; ‘si-NC’ refers to normal control siRNA

Following the transfection of Rab25 siRNA, the expression levels of Rab25 protein in LNCaP cells were significantly decreased (P = 0.009, Fig. 4a). As shown in Fig. 4b, c, the cell proliferation, invasion and migration abilities of LNCaP cells with Rab25 siRNA transfection were dramatically lower than those with control siRNA transfection (all P < 0.05).

## Discussion

In the current study, the expression of Rab25, at both mRNA and protein levels, were evaluated based on a large cohort of PCa patients using paired PCa and adjacent normal prostate tissues. Our data not only confirmed the Rab25 specific overexpression in PCa tissues, but also revealed its cytoplasmic localization in PCa cells. Importantly, high expression of Rab25 protein was dramatically associated with high Gleason score, the presence of distant metastasis, poor overall survival and biochemical recurrence-free survival. To the best of our knowledge, these findings imply for the first time that Rab25 might be a potential marker for early diagnosis and unfavorable prognosis in PCa patients.

Ras-related protein 25, as a member of Rab GTPases that are the largest subfamily of small GTPases, contains a different GTP-binding sequence (WDTAGLE), which is different from other Rab members [22]. Rab GTPases play a role in regulating intracellular vesicle transport and protein trafficking, and in maintaining epithelial cell polarity program, which are essential hallmarks of cancer [23]. The involvement of Rab25 in human cancer was originally reported by He et al. [24] who found the upregulation of six human Rab GTPases in liver cancer. After that, accumulating studies have revealed the aberrant expression of Rab25 and its associations with development, progression and prognosis of numerous types of human cancers. For example, Cao et al. [9] showed the overexpression of Rab25 protein in gastric cancer compared to adjacent normal gastric tissues by immunohistochemistry and Western blot, and also confirmed that it promoted the invasion and metastasis of gastric cancer; Mitra et al. [15] substantiated a striking context dependent role of Rab25 in breast cancer where Rab25 may be amplified and enhanced aggressiveness in luminal B cancers while in claudin-low tumors, Rab25 may be lost implying possible anti-tumor functions; Zhang et al. [12] indicated that Rab25 may promote metastasis of bladder cancer via inducing the epithelial-mesenchymal-transition process and activating Akt/GSK-3β/Snail signaling pathway; Li et al. [13] revealed that high levels of Rab25 expression were correlated with renal cell carcinoma invasion classification, lymph-node metastasis and pathological stage; Liu et al. [20] also identified Rab25 as a potential prognostic biomarker in clear cell renal cell carcinoma; Geng et al. [25] found that Rab25 was overexpressed in human hepatocellular carcinoma and may contribute to cancer cell proliferation and invasion possibly through regulation of the Wnt signaling pathway; Ma et al. [26] indicated that Rab25 expression was a potential prognostic index for advanced non-small cell lung cancer patients and its inhibition might improve chemosensitization in non-small cell lung cancer treatment. In contrast, the data of Te′llez-Gabriel et al. [14] supported a tumor suppressor role for Rab25 in head and neck squamous cell carcinoma and its potential use to identify locally advanced patients with a high probability of survival after genotoxic treatment; Goldenring et al. [16] reported that colorectal adenocarcinomas with low Rab25 correlated with shorter patient survival. In the current study, our data demonstrated that Rab25 overexpression was associated with malignant clinicopathologic characteristics and poor prognosis in PCa patients. These findings suggest the context dependent roles of Rab25 in various human cancers which may be caused by the heterogeneity of carcinogenesis in different tissues.

Since Rab25 mRNA and protein expression in PCa tissues and cells were distinctly higher than those in non-cancerous prostate tissues and normal prostate epithelial cells, and its overexpression associated with aggressive clinical progression and poor patients’ prognosis, we hypothesized Rab25 might function as an oncogene in PCa. Therefore, we investigated whether the knockdown of Rab25 in PCa cells could influence the abilities of cell proliferation, invasion and migration. The results showed that cell growth, invasion and migration were all inhibited in PCa cells transfected with Rab25 siRNA compared with the cells transfected with control siRNA.

## Conclusions

Our data offer the convincing evidence that high expression of Rab25 may contribute to malignant progression and biochemical recurrence of PCa patients after radical prostatectomy. The exact mechanisms must be further studied.

## Additional file

**Additional file 1: Figure S1.** Representative image of Rab25 protein immunostaining in a RCC tissue.

## Abbreviations

PCa: prostate cancer
PSA: prostate-specific antigen
GS: Gleason score
Rab25: Ras-related protein 25
CT: cycle threshold
SDS-PAGE: sodium dodecyl sulfate polyacrylamide gel electrophoresis
PBS: phosphate buffered saline
DAB: 3,3′-diaminobenzidine tetrahydrochloride
IRS: immunoreactive score
