# Supplementary figures and images for: High expression of Rab25 contributes to malignant phenotypes and biochemical recurrence in patients with prostate cancer after radical prostatectomy
Source: Cancer Cell Int. 2017 Apr 11;17:45. doi: 10.1186/s12935-017-0411-0 (PMC5387234; doi:10.1186/s12935-017-0411-0)

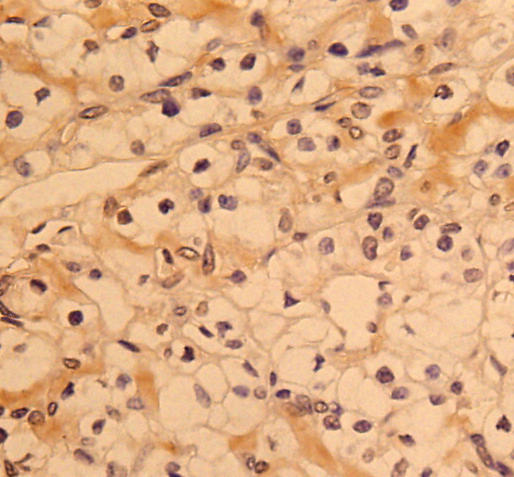


**Supplementary Figure S1.**

**Representative image of Rab25 protein immunostaining in a RCC tissue.**

Supplement: Supplementary file 1 — Additional file 1: Figure S1. Representative image of Rab25 protein immunostaining in a RCC tissue. [file 12935_2017_411_MOESM1_ESM.docx]
